# Environmentally-relevant concentrations of the antipsychotic drugs sulpiride and clozapine induce abnormal dopamine and serotonin signaling in zebrafish brain

**DOI:** 10.1038/s41598-022-22169-1

**Published:** 2022-10-26

**Authors:** Bo Zhang, Xijian Peng, Xiumei Sun, Yuanming Guo, Tiejun Li

**Affiliations:** 1 The Key Laboratory of Environmental Pollution Monitoring and Disease Control, Ministry of Education, School of Public Health, Guizhou Medical University, Guiyang, 550025 Guizhou China; 2 Marine Fisheries Research Institute of Zhejiang, Zhoushan, 316021 China; 3 Marine and Fishery Institute of Zhejiang, Ocean University, Zhoushan, 316021 China

**Keywords:** Environmental impact, Risk factors

## Abstract

The presence of drugs in surface and groundwaters adversely affects the physiological function of non-target organisms due special activities that can pose a serious threats to various forms of aquatic life. Psychotropic drugs are one of the most commonly used drugs in the world. Hence, the aim of this study was to investigate the effect of environmentally-relevant concentrations of the antipsychotic drugs, sulpiride and clozapine, on dopaminergic (DAergic) and serotonergic (5-HTergic) neurotransmitter systems in the brain of zebrafish. Adult zebrafish (AB strain) were exposed to the environmentally-relevant concentrations of sulpiride, clozapine, or a mixture of sulpiride and clozapine. The effects of the drugs on the mRNA and protein levels of major functional molecules in DAergic and 5-HTergic systems were then analyzed in the telencephalon and diencephalon. Both drugs induced abnormal mRNA and protein levels of important functional molecules of the DA and 5-HT signaling pathways in both telencephalon and diencephalon, as shown by the abnormal transcriptional levels of *TH*, *DAT*, *DR D1*, *DR D2*, *MAO*, *TPH*, *serotonin transporter (SERT)*, *5-HTR 1AA*, *5-HTR 1B*, *5-THR 2AA*, and *5-HTR 2B*, and the abnormal translational levels of DAT, DR D2, SERT, 5-HTR 1A, 5-HTR 1B, and 5-HTR 2B. In addition, we observed a specificity in the adverse effects of these antipsychotic drugs, in terms of doses and brain parts. Compared to their effects alone, the drug mixture had a weaker effect on the DA and 5-HT systems, suggesting an antagonistic interaction between sulpiride and clozapine. Our findings suggest that sulpiride and clozapine interfere with DAergic and 5-HTergic neurotransmitter systems in the telencephalon and diencephalon of zebrafish, resulting in possible effects on brain functions and posing a serious threat to the health of zebrafish.

## Introduction

In the past 20 years, various drugs have been found in a variety of aquatic environments, such as domestic sewage, surface waters, and groundwater, throughout the world, including North America, Europe, and some countries in Asia^1,2^. Although the concentrations of drugs in the aquatic environment are very low (in the order of ng/L), they pose a serious threat to the health of non-target aquatic organisms because of their special activities that have adverse impacts on the physiological function of organisms^3^. Therefore, the effects of environmental pollution of water bodies on the health of aquatic organisms has become a subject of intense research in ecotoxicology.

Schizophrenia is a complex neuropsychiatric syndrome that comprises a group of severe mental illnesses of unknown etiology. The basis for the pathology of schizophrenia is the hyperfunction of the dopamine (DA) system in the mesolimbic and mesocortical pathways, accompanied by metabolic disorders of serotonin (5-hydroxytryptamine, 5-HT). Therefore, the first and second generations of antipsychotic drugs (typical and atypical, respectively), were designed and produced to address this pathology. The first-generation of antipsychotic drugs mainly act on the dopamine D2 receptors (DR D2) of the central nervous system, i.e., they act as a DR D2 receptor antagonist. The second-generation of antipsychotic drugs mainly act on 5-HT2 receptors (5-HTR2) of the central nervous system, with high and low affinities for 5-HTR2 and DR D2, respectively. Sulpiride {( ±)-5-(amino sulfonyl)-N-[(1-ethylpyrrolidin-2-yl) methyl]-2-methoxybenzamide} is a first-generation antipsychotic drug of the benzamide class, while clozapine {8-chloro-11-(4-methylpiperazin-1-yl) -5H-dibenzo [b, e]^[1,4]^ diazepine} is a second-generation antipsychotic drug of the dibenzodiazepine class.

The impact of drugs on the organism is mainly due to their biological activity and environmental persistence. The pharmacological action of drugs is based on their action on specific cellular molecular targets that interrupt certain physiological functions in organisms. As these molecular sites are often highly conserved, various species are often susceptible to the adverse effects of drugs. Relatively low concentrations of drugs are continuously flowing into the surface water, groundwater, and ocean. Therefore, aquatic organisms, especially those near the coastline, are chronically exposed to these drugs, which tend to bioaccumulate with serious effects, often threatening the health of aquatic organisms^4^.

The DA neurotransmitter system in the fish brain plays an important role in regulating movement, motivation, behavior, learning, and memory^5,6^. Originating from the periventricular nucleus of the posterior tuberculum, the dorsal (TPpd) and ventral (TPpv) subdivisions of dopaminergic fibers of the diencephalon are distributed in the lateral part of the dorsal telencephalon (pallium), the anterior–posterior commissure, and the dorsal part of the ventral telencephalon^7,8^. As a neurotransmitter, DA can be synthesized by tyrosine hydroxylase (TH) and be released from pre-synapse terminal into synaptic cleft to act on post-synaptic dopaminergic (DAergic) receptors (DR), and can be re-uptake by dopamine transporter (DAT) or be metabolized by monoamine oxidase (MAO). Four classes of DR have been identified in zebrafish, and these correspond to the mammalian DR D1, DR D2, DR D3, and DR D4 orthologs^9,10^. In zebrafish, DR D1 and DR D2 are involved in regulating associative learning^11^, with DR D2 regulating the process of acquisition and consolidation of spatial information in latent learning^12^. The serotonergic (5-HTergic) system in zebrafish brain may be involved in regulating fear/anxiety, stress, aggression, defensive behavior^13,14^. Similar to DA, 5-HT is synthesized by tryptophan hydroxylase (TPH) and re-uptake by serotonin transporter (SERT). A total of 6 types 5-HT receptors (5-HTR) have been identified in zebrafish, and these receptors are homologous to the 5-HTRs in humans and mice^15^. The results of previous studies show that 5-HTR1A, 5-HTR1B, and 5-HTR2 are involved in regulating zebrafish anxiety-like and defense behavior^13,16^. 5-HTergic neurons in the dorsal raphe nucleus (Rd) in zebrafish brain projects to the telencephalic regions, and are mainly distributed in the lateral parts of the dorsal and ventral telencephalon, as well as in the diencephalic area, which may modulate the activity of the DAergic systems in the telencephalon and diencephalon^13,17^.

Antipsychotic drugs can flow continuously into the aquatic environment through a variety of routes, threatening the health of aquatic organisms. To date, toxicological research on antipsychotic drugs have focused mainly on their side effects on human health. Studies on the adverse effects of antipsychotic drugs polluting the environment on the health of aquatic organisms are very scarce.

The aim of this study is to explore the effects of environmentally-relevant concentrations of antipsychotic drugs on DAergic and 5-HTergic systems in the brain of zebrafish. The results of this study will provide a scientific basis for conducting more detailed studies on the effects of antipsychotic drugs on the physiological function of zebrafish brain.

## Materials and methods

### Chemicals

Sulpiride (S8010, CAS:15676-16-1) and clozapine (C6305, CAS:5786-21-0) were obtained from Sigma-Aldrich Co. LLC (USA). Both drugs were dissolved in 1% ethanol and then diluted with distilled water to prepare stock solutions with a final concentration of 1 mg/L. The solutions were stored at 4 °C protected from light.

### Animal maintenance and treatments

Adult zebrafish (AB strain, 4-month-old) were provided by the Center for Excellence in Molecular Cell Science, Chinese Academy of Sciences. Zebrafish were housed in a light- and temperature-controlled aquaculture facility with a standard 14:10 h light/dark photoperiod and a constant temperature of 26 ± 1 °C. The fish were fed with brine shrimp spawn twice daily.

Antipsychotic drug concentrations were based on the median concentrations measured in surface waters and reported in previous studies^18,19^. Each group of zebrafish (30 per group with a total of 10 groups) were treated with increasing (20 ng/L, 50 ng/L, and 100 ng/L) concentrations of sulpiride, clozapine, and a mixture of sulpiride and clozapine (1:1, to explore the interaction between two drugs based the pharmacologic action) for periods of 14 days for each concentration, and a 0.01% ethanol solution was used for the control. Half of the water in each experimental tank was replaced every day with dechlorinated water containing the corresponding concentrations of antipsychotic drug(s). All animals in this study were treated in accordance with the criteria outlined in the Guide for the Care and Use of Laboratory Animals prepared by the National Institutes of Health. All experiments were assessed and approved by the Ethics Committee of Zhejiang Ocean University. All methods are reported in accordance with ARRIVE guidelines.

### Total RNA extraction and qRT-PCR

At the end of the treatments, the whole brain of 10 zebrafish for each group were collected after anesthetizing the fish with MS-222 (Sigma-Aldrich Co. LLC, USA). Each brain was separated into the telencephalon and diencephalon, and total RNA was extracted from each, separately. RNA was extracted using the RNAeasy ™ animal RNA extraction kit (Beyotime, Shanghai, China) according to the manufacturer’s instructions. In brief, 15–20 mg of brain was placed in a 1.5-ml centrifuge tube, and mixed with 600 μl of ice-cooled lysis buffer and homogenized with a micro-electric homogenizer. The homogenate was centrifuged at approximately 14,000×*g* for 2 min and the supernatant was mixed with an equal volume of binding solution. The mixture was gently inverted 3–5 times, and then transferred into a purification column. The column was centrifuged at 12,000×*g* for 30 s, and then it was washed with 600 μl each of wash solutions I and II by centrifuging at 12,000×*g* for 30 s. The washed column was then centrifuged at approximately 14,000–16,000×*g* for 2 min to remove all residual liquid. The RNA was eluted with 30–50 μl of elution buffer, left at room temperature for 2–3 min then centrifuged at the highest speed for 30 s. The solution was the purified RNA. RNA quality was determined by NANODROP 2000c. Reverse transcription was performed using the BeyoRT™ II cDNA Synthesis Kit (Beyotime, Shanghai, China) using approximately 1 μg of RNA.

We explored the effects of antipsychotics on the function of DAergic and 5-HTergic neurotransmitter systems of telencephalon and diencephalon in zebrafish by looking at the following genes: important functional molecules in the DA system included the DA synthesis key enzyme, *TH*, as well as *DAT* and the *DR* (*DR D1* and *DR D2*). In the 5-HT system we looked at the 5-HT synthetic key enzyme *TPH*, as well as *SERT*, the *5-HTR* (*5-HTR 1AA*, *5-HTR 1B*, *5-THR 2AA*, *5-HTR 2B*), and *MAO*. The primer sequences for the genes encoding these enzymes, transporters, and receptors are shown in Table 1. Q-PCR amplifications were carried out in a CFX connect detection system (Bio-rad, USA) using the BeyoFast™ SYBR Green qPCR Mix (Beyotime, Shanghai, China). All PCRs were performed with three replicates. The amplification cycles consisted of: 2 min at 95 °C, 40 cycles of 15 s at 95 °C and 30 s at 60 °C. Melting curve analysis was performed to check the specificity of the primers. Expression data was normalized against the expression of *β-actin*. The relative quantity of each gene transcript was calculated using the 2^−∆∆Ct^ method^20^^.^

**Table 1:** Primer sequences.

Genes	Primer type	Sequences
*TH*	Forward	TGAAACCAGACCCAGCCGAAAAC
	Reverse	CCAGCGTGCTAACATCCGACAG
*DAT*	Forward	CCGCTCTACGCCTTCTACAAGTTC
	Reverse	AGGTGATGGTCAGTCTCAGGAGTG
*DR D1*	Forward	CTGCTGACGGACAACTGTGACTC
	Reverse	CGTATCTGCTTCTGGGCGATTCTG
*DR D2*	Forward	AGCAGGATCAGTCTGGTGGAAGTC
	Reverse	GCTTCAGGCGATGGAGGTGATATG
*MAO*	Forward	TGGCGGAGGCATCTCAGGTC
	Reverse	TTCTTCCACCAACACGGCTTCTG
*TPH*	Forward	CTGCGACTCCCGTGAAGACAAC
	Reverse	GCCTGATGGTGAAGCCTGTTCTC
*SERT*	Forward	TAGATCGGCACACCCATGAGTCC
	Reverse	CCCAGAGCAATAAGGAGCAAGACG
*5-HTR 1AA*	Forward	GTCATCGCTGCTATCGCCTTGG
	Reverse	GCACCAGCACCGACACCATAAG
*5-HTR 1B*	Forward	TGCGTTTGTCATTGCCACCATTTC
	Reverse	CACGAGCACCGACACCAGAAG
*5-THR 2AA*	Forward	CAACGGCGAGACGACCAAAGAG
	Reverse	AGCCACCGTAACAATGACCACAAC
*5-HTR 2B*	Forward	TTTCAGTGGGTGGGCTATGTTTCG
	Reverse	TCGGAGTCCTCACGCTCTTGTAG
*β-actin*	Forward	GTGATGGACTCTGGTGATGGTGTG
	Reverse	CACGCTCGGTCAGGATCTTCATC

### Western blot analysis

After collecting the whole brain of zebrafish (20 for each group), we extracted the total proteins of the telencephalon and diencephalon, separately. Brain tissues were washed 2–3 times with ice-cold PBS, 10x, then an equal volume of tissue lysis buffer (P0033, Beyotime Institute of Biotechnology, Shanghai, China) was added (protease inhibitors were added within a few minutes before use) and the tissue was homogenized. The homogenate was placed on ice for 30 min, with shaking every 5 min to ensure the complete lysis of the tissue. The lysate was centrifuged at 12,000×*g* at 4 °C for 10 min. The supernatant containing the total proteins was collected and the protein concentration was determined using a BCA kit (Beyotime Institute of Biotechnology). Equal amounts of protein (30 μg) were separated on 10% sodium dodecyl sulfate–polyacrylamide gels and then transferred onto polyvinylidene difluoride membranes. The membranes were blocked with 5% skimmed milk powder for 30 min and then incubated with rabbit anti-DAT (dilution, 1:1000; Invitrogen, PA5-78382), goat anti-DR D2 (dilution, 1:1000; abcam, ab30743), rabbit anti-SERT (dilution, 1:1000; abcam, ab272912), rabbit anti-5-HTR 1A (dilution, 1:1000; abcam, ab227165), rabbit anti-5-HTR 1B (dilution, 1:500; Invitrogen, PA5-111905), rabbit anti-5-HTR 2B (dilution, 1:700; Solarbio, K004123P) and mouse anti-β-actin (dilution, 1:3000; WUHAN SANYING, 66009-1-lg) at 4 °C overnight. This was followed by an incubation with horseradish peroxidase goat anti-rabbit, donkey anti-goat or goat anti-mouse IgG (dilution, 1:5000; Beyotime, Shanghai, China) for 30 min. The ECL kit (Beyotime, Shanghai, China) was used to detect immunoreactive signals, which were then scanned using a scanner (EPSON, V370). Band intensities were measured using AlphaEaseFC (Alpha Innotech) software. The Western blot analysis was repeated three times.

### Statistical analysis

Results are presented as means ± standard errors of the mean. Differences in the relative levels of transcripts of *TH*, *DAT*, *DRD1*, *DRD2*, *MAO*, *TPH*, *SERT*, *5-HTR1AA*, *5-HTR1B*, *5-THR2AA*, and *5-HTR2B*, and differences in the band intensities of DAT, DR D2, SERT, 5-HTR 1A, 5-HTR 1B, and 5-HTR 2B among different groups were analyzed using ANOVA followed by t-tests (data were normalized to β-actin prior to statistical analysis). All statistical analyses were performed with SPSS 20.0 and Graph Pad Prism 8.0 software. A probability (*p*) value < 0.05 was considered statistically significant.

### Ethical approval

All experiments were assessed and approved by the Ethics Committee of Zhejiang Ocean University. All methods are reported in accordance with ARRIVE guidelines.

## Results

### Effects of sulpiride and clozapine on mRNA levels of functional genes for DAergic and 5-HTergic systems in zebrafish telencephalon

**Figure 1:**
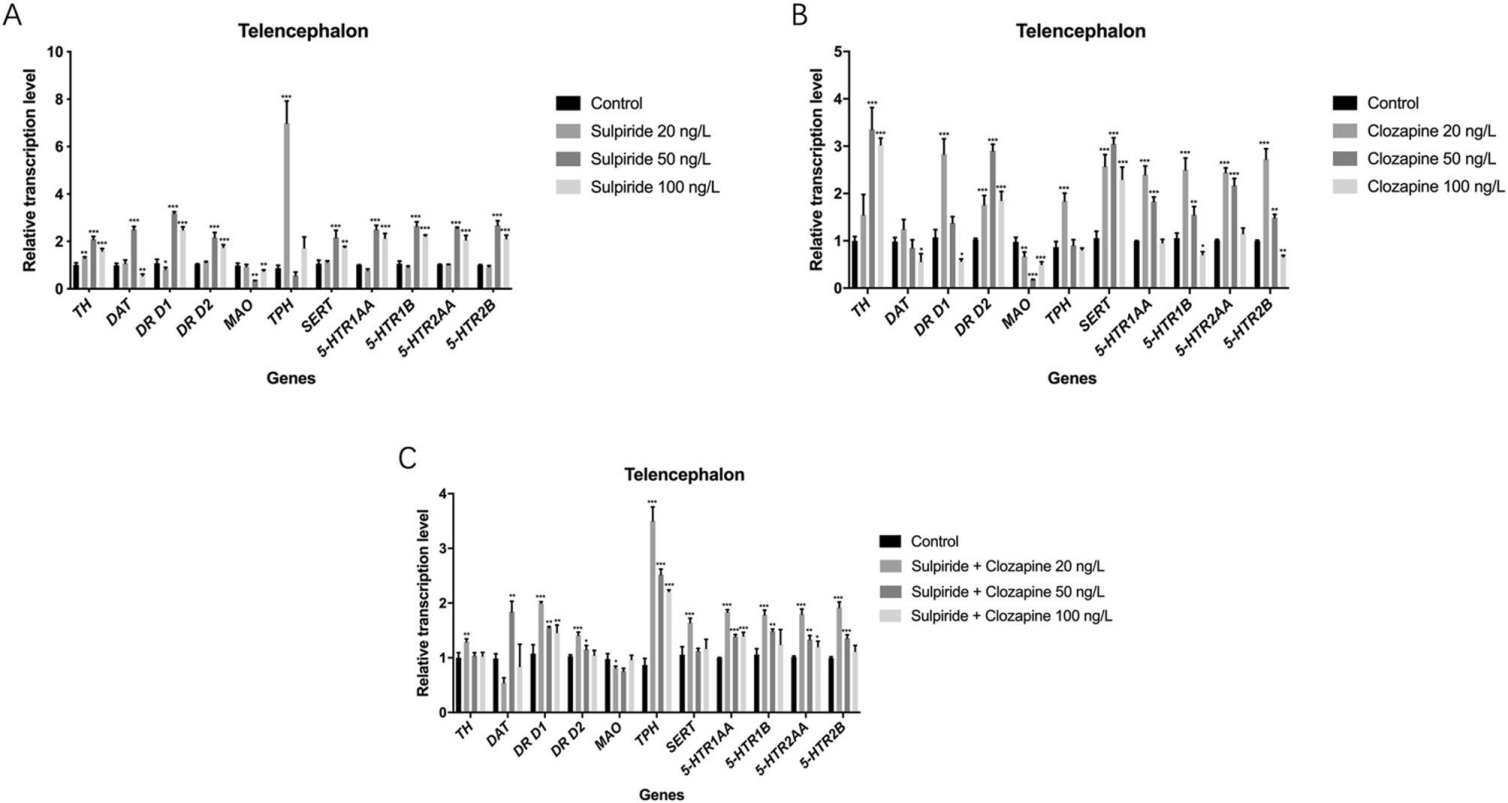
Changes in the mRNA levels of functional molecules involved in DAergic and 5-HTergic systems in zebrafish telencephalon induced by sulpiride and clozapine. Note: (**A**) Sulpiride, (**B**) Clozapine, (**C**) Mixture of sulpiride and clozapine. Data are presented as means ± SE (n = 10). *p* < 0.05 was considered statistically significant. *p < 0.05, **p < 0.01, ***p < 0.001.

Sulpiride-induced abnormal transcriptional levels of *TH*, *DAT*, *DR D1*, *DR D2*, *MAO*, *TPH*, *SERT, 5-HTR 1AA*, *5-HTR 1B*, *5-THR 2AA*, and *5-HTR 2B* in zebrafish telencephalon (*p* < 0.001). The mRNA level of *TH* (*p* < 0.01) was up-regulated while that of *DR D1* (*p* < 0.05) was down-regulated by 20 ng/L sulpiride. The mRNA expression levels of *TH*, *DAT*, *DR D1*, and *DR D2* (*p* < 0.001) were up-regulated by 50 ng/L sulpiride; *TH*, *DR D1*, and *DR D2* transcripts (*p* < 0.001) were also up-regulated, while that of *DAT* was down-regulated by 100 ng/L sulpiride (Fig. 1). In addition, sulpiride up-regulated the mRNA expression levels of *TPH*, *SERT*, *5-HTR 1AA*, *5-HTR 1B, 5-THR 2AA*, and *5-HTR 2B* (*p* < 0.001), while it down-regulated *MAO* mRNA expression level (*p* < 0.001) (Fig. 1).

**Figure 2:**
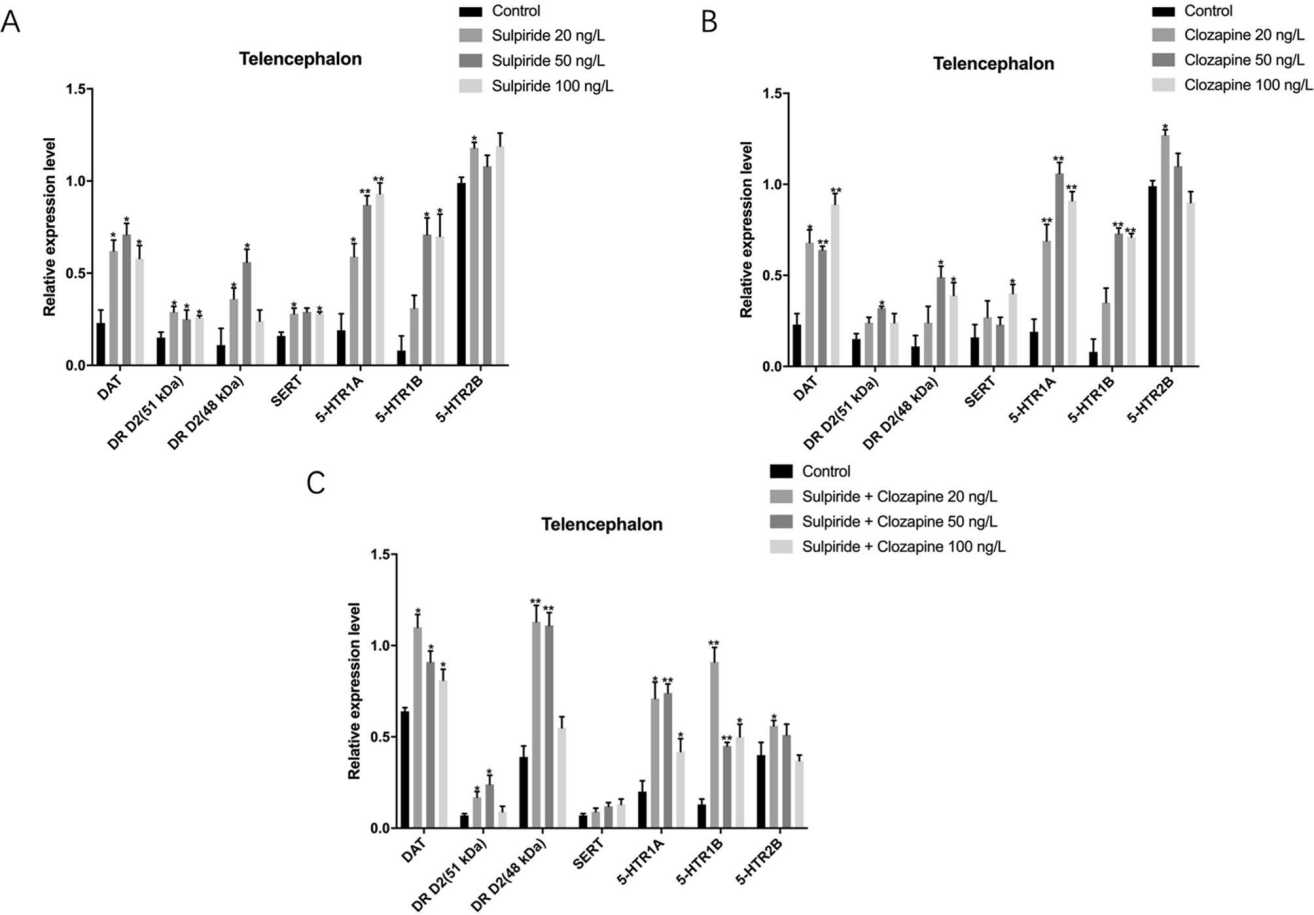
Changes in the protein levels of functional molecules involved in DAergic and 5-HTergic systems in zebrafish telencephalon induced by sulpiride and clozapine. Note: (**A**) Sulpiride, (**B**) Clozapine, (**C**) Mixture of sulpiride and clozapine. Data are presented as means ± SE (n = 20). *p* < 0.05 was considered statistically significant. *p < 0.05, **p < 0.01.

Clozapine caused abnormal transcriptional levels of *TH*, *DAT*, *DR D1*, *DR D2*, *MAO*, *TPH*, *SERT, 5-HTR 1AA*, *5-HTR 1B*, *5-THR 2AA*, and *5-HTR 2B* in zebrafish telencephalon (*p* < 0.001). The results show that clozapine significantly increased the expression of *TH*. The mRNA expression levels of *DAT* (*p* > 0.05), *DR D1* (*p* < 0.001), and *DR D2* (*p* < 0.001) were up-regulated by 20 ng/L clozapine; while those of *DR D1* (*p* > 0.05) and *DR D2* (*p* < 0.001) were up-regulated in 50 ng/L clozapine; while those of *DAT* (*p* < 0.05) and *DR D1* (*p* < 0.05) were down-regulated and those of *DR D2* (*p* < 0.001) were up-regulated by 100 ng/L clozapine (Fig. 2). Moreover, clozapine induced the up-regulation of the mRNA expression levels of *TPH*, *SERT*, *5-HTR 1AA*, *5-HTR 1B, 5-THR 2AA*, and *5-HTR 2B* (*p* < 0.001), although 100 ng/L clozapine inhibited the mRNA expression levels of *5-HTR 1B* (*p* < 0.05), *5-HTR 2B* (*p* < 0.01), and *MAO* (*p* < 0.001) (Fig. 1).

The sulpiride and clozapine mixture significantly affected the mRNA expression levels of *TH*, *DAT*, *DR D1*, *DR D2*, *MAO*, *TPH*, *SERT, 5-HTR 1AA*, *5-HTR 1B*, *5-THR 2AA*, and *5-HTR 2B* in zebrafish telencephalon (*p* < 0.01). All concentrations of the mixture increased the mRNA expression levels of *TH*, *DR D1*, and *DR D2* (*p* < 0.01). At 20 ng/L, the mixture decreased the mRNA of *DAT* (*p* > 0.05), while increasing that of *DAT* at 50 ng/L (*p* < 0.01) (Fig. 1). In addition, all concentrations of the mixture up-regulated the mRNA expression levels of *TPH*, *SERT*, *5-HTR 1AA*, *5-HTR 1B, 5-THR 2AA*, *5-HTR 2B* (*p* < 0.01), while down-regulating that of *MAO* (*p* < 0.01) (Fig. 1).

### Effects of sulpiride and clozapine on the translation levels of functional proteins involved in DAergic and 5-HTergic systems in zebrafish telencephalon

**Figure 3:**
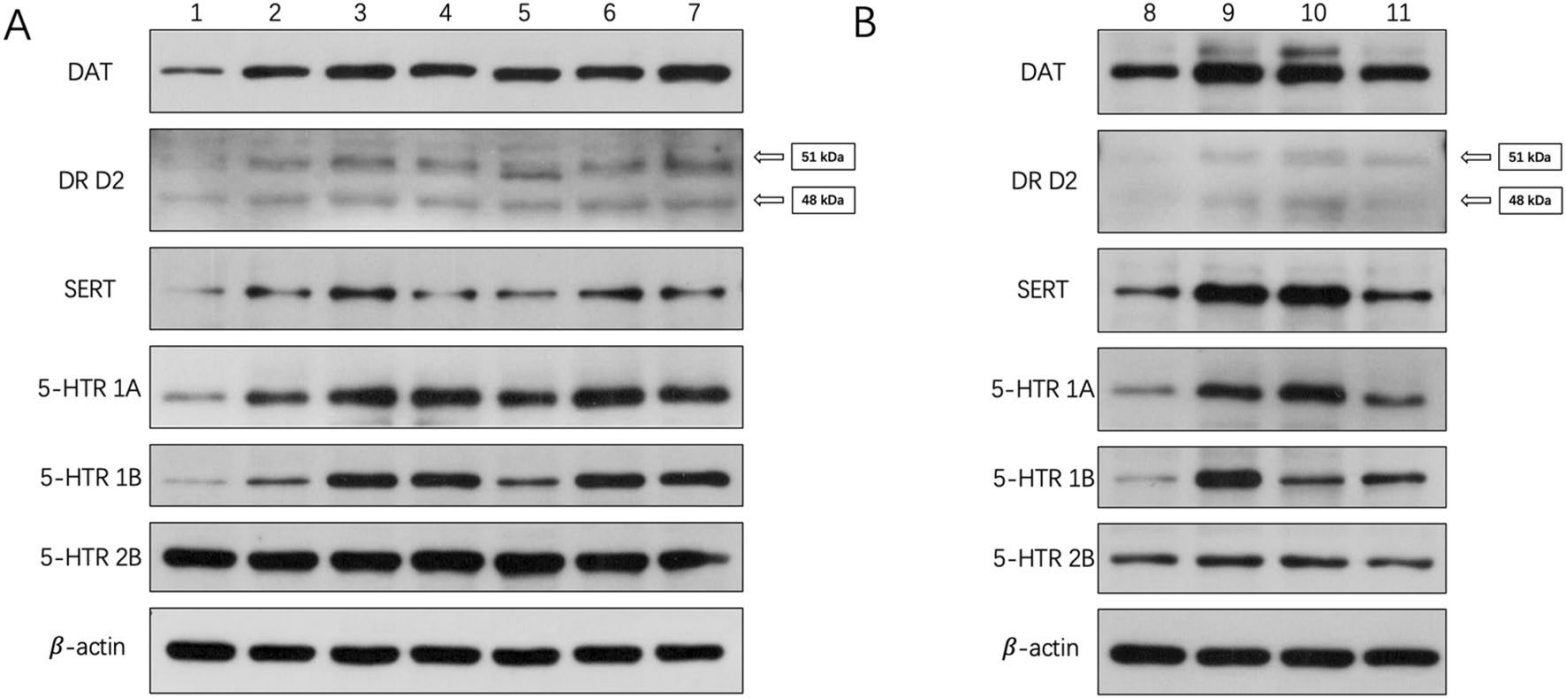
Effects of sulpiride and clozapine on the translation levels of functional proteins involved in DAergic and 5-HTergic systems in zebrafish telencephalon. Lane 1, control; lanes 2–4, 20, 50, and 100 ng/L sulpiride, respectively; lanes 5–7, 20, 50, and 100 ng/L clozapine, respectively; lane 8, control; lanes 9–11, 20, 50, and 100 ng/L sulpiride and clozapine mixture, respectively. The grouping of blots was first cropped from different gels and then transferred onto PVDF membranes (1:1) prior to hybridisation with antibodies.

Sulpiride-induced abnormal protein levels of DAT, DR D2 (51 kDa), DR D2 (48 kDa), SERT, 5-HTR 1A, 5-HTR 1B, and 5-HTR 2B in zebrafish telencephalon (*p* < 0.05). Compared to the control, sulpiride significantly increased the protein levels of DAT, DR D2 (51 kDa), DR D2 (48 kDa), SERT, 5-HTR 1A, 5-HTR 1B, and 5-HTR 2B (Figs. 2, 3).

Clozapine-induced abnormal protein levels of DAT, DR D2 (51 kDa), DR D2 (48 kDa), SERT, 5-HTR 1A, 5-HTR 1B, and 5-HTR 2B in zebrafish telencephalon (*p* < 0.05). Specifically, all concentrations of clozapine up-regulated DAT, DR D2 (51 kDa), DR D2 (48 kDa), 5-HTR 1A, and 5-HTR 1B (Figs. 2, 3). Meanwhile, the protein levels of 5-HTR 2B and SERT were increased by both 20 ng/L and 100 ng/L clozapine (*p* < 0.05) (Figs. 2, 3).

All concentrations of sulpiride and clozapine mixture significantly affected the protein levels of DAT, DR D2 (51 kDa), DR D2 (48 kDa), SERT, 5-HTR 1A, and 5-HTR 1B in zebrafish telencephalon (*p* < 0.05). Specifically, the mixture up-regulated DAT, DR D2 (51 kDa), DR D2 (48 kDa), 5-HTR 1A, and 5-HTR1B compared to the control (Figs. 2, 3). In addition, the effects of exposure of the DAergic and 5-HTergic systems of zebrafish telencephalon to the mixture decreased with decreasing mixture concentrations, as seen in the decreasing trend in the protein levels of functional molecules of the two neurotransmitter systems (Figs. 2, 3). Moreover, protein level of 5-HTR 2B was up-regulated by 20 ng/L mixture (*p* < 0.05) (Figs. 2, 3).

### Effects of sulpiride and clozapine on mRNA levels of functional genes involved in DAergic and 5-HTergic systems in zebrafish diencephalon

**Figure 4:**
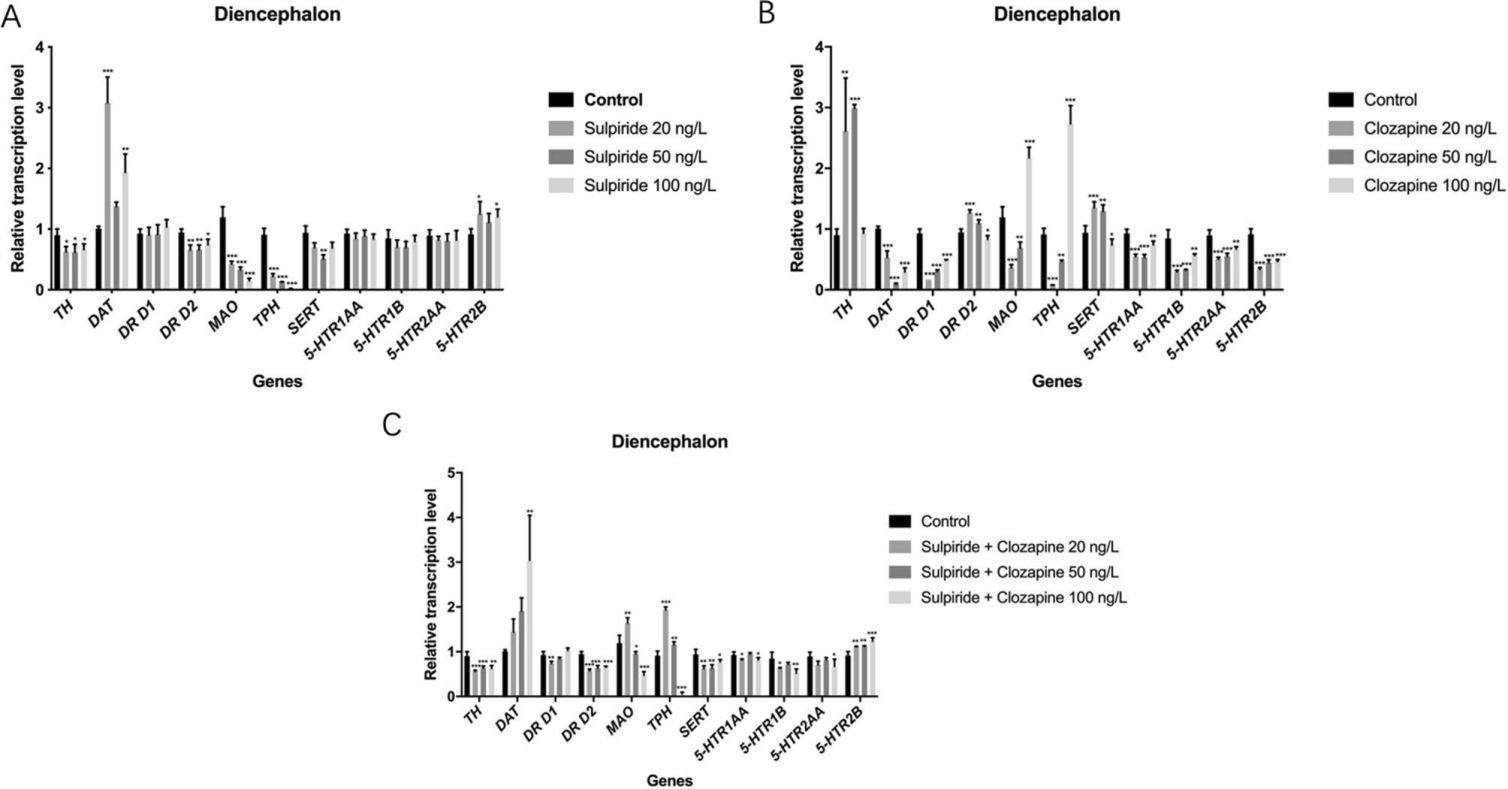
Changes in the mRNA levels of functional molecules involved in DAergic and 5-HTergic systems in zebrafish diencephalon induced by sulpiride and clozapine. Note: (**A**) Sulpiride, (**B**) Clozapine, (**C**) Mixture of sulpiride and clozapine. Data are presented as means ± SE (n = 10). *p* < 0.05 was considered statistically significant. *p < 0.05, **p < 0.01, ***p < 0.001.

Sulpiride-induced abnormal mRNA expression levels of *TH*, *DAT*, *DRD2*, *MAO*, *TPH*, *SERT* in zebrafish diencephalon (*p* < 0.05). Specifically, sulpiride down-regulated the mRNA expression levels of *TH* (*p* < 0.05), *DR D2* (*p* < 0.01), *TPH* (*p* < 0.001), *SERT* (*p* < 0.05), and *MAO* (*p* < 0.001), while up-regulating those of *DAT* (*p* < 0.001) and *5-HTR 2B* (*p* > 0.05) (Fig. 4).

Clozapine caused abnormal transcriptional levels of *TH*, *DAT*, *DR D1*, *DR D2*, *MAO*, *TPH*, *SERT, 5-HTR 1AA*, *5-HTR 1B*, *5-THR 2AA*, and *5-HTR 2B* in zebrafish telencephalon (*p* < 0.001). The mRNA expression levels of *TH* (*p* < 0.01), *DR D2* (*p* < 0.001) were up-regulated, while those of *DAT* (*p* < 0.001) and *DR D1* (*p* < 0.001) were down-regulated by 20 ng/L clozapine. *TH* (*p* < 0.001) and *DR D2* (*p* < 0.01) were up-regulated, while *DAT* and *DR D1* (*p* < 0.001) were down-regulated by 50 ng/L clozapine. The mRNA expression levels of *DAT* (*p* < 0.001)*, DR D1* (*p* < 0.001) and *DR D2* (*p* < 0.05) were significantly inhibited by 100 ng/L clozapine (Fig. 4). Moreover, both 20 ng/L and 50 ng/L clozapine down-regulated *TPH, 5-HTR 1AA*, *5-HTR 1B*, *5-THR 2AA*, and *5-HTR 2B* (*p* < 0.001), while up-regulating *SERT* mRNA level (*p* < 0.001). However, 100 ng/L clozapine for up-regulated *TPH* (*p* < 0.001), *SERT*, *5-HTR 1AA*, *5-HTR 1B*, and *5-THR 2AA*, while down-regulating *5-HTR 2B* (*p* < 0.05). Furthermore, *MAO* was down-regulated by both 20 ng/L and 50 ng/L clozapine (*p* < 0.05), while being up-regulated by 100 ng/L clozapine (*p* < 0.001) (Fig. 4).

The sulpiride and clozapine mixture significantly affected the mRNA expression levels of *TH*, *DAT*, *DR D1*, *DR D2*, *MAO*, *TPH*, *SERT, 5-HTR 1AA*, *5-HTR 1B*, *5-THR 2AA*, and *5-HTR 2B* in zebrafish telencephalon (*p* < 0.05). Specifically, all concentrations of the mixture down-regulated *TH*, *DR D1*, and *DR D2*, while up-regulating *DAT* (*p* < 0.01). In addition, the mixture decreased the mRNA expression levels of *SERT*, *5-HTR 1AA*, *5-HTR 1BTH*, *DR D1*, and *DR D2* (*p* < 0.01), while increasing that of *5-HTR 2B* (*p* < 0.05). *TPH* was up-regulated by 20 ng/L and 50 ng/L mixture (*p* < 0.05), while it was down-regulated by the 100 ng/L mixture (*p* < 0.001) (Fig. 4). In addition, *MAO* was up-regulated by the 20 ng/L mixture (*p* < 0.01), while it was down-regulated by the 50 ng/L and 100 ng/L mixtures (*p* < 0.05). Furthermore, *5-HTR 2AA* was down-regulated by the 100 ng/L mixture (*p* < 0.05) (Fig. 4).

### Effects of sulpiride and clozapine on the translation levels of functional proteins involved in DAergic and 5-HTergic systems in zebrafish diencephalon

**Figure 5:**
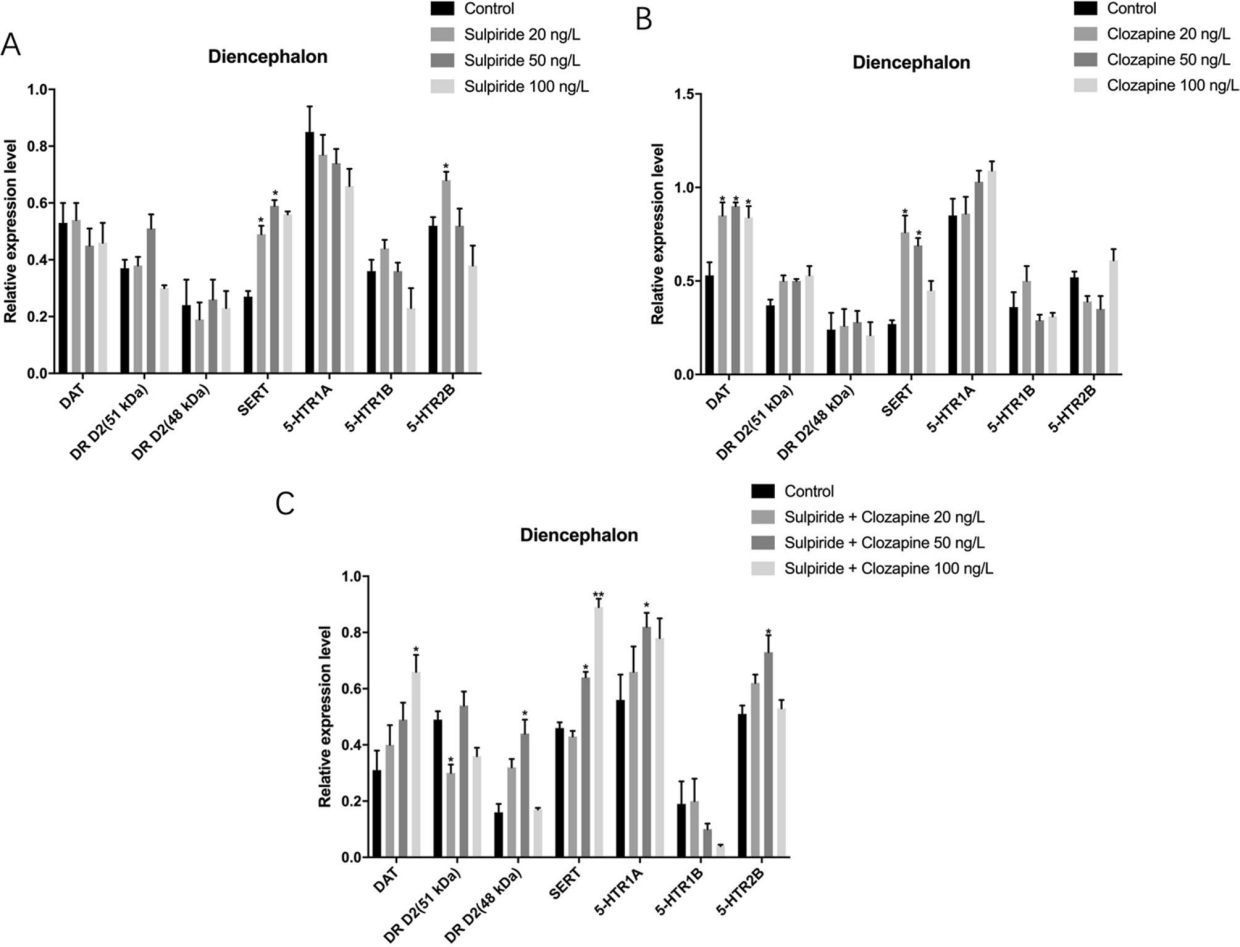
Changes in the protein levels of functional molecules involved in DAergic and 5-HTergic systems in zebrafish diencephalon induced by sulpiride and clozapine. Note: (**A**) Sulpiride, (**B**) Clozapine, (**C**) Mixture of sulpiride and clozapine. Data are presented as means ± SE (n = 20). *p* < 0.05 was considered statistically significant. *p < 0.05, **p < 0.01.

**Figure 6:**
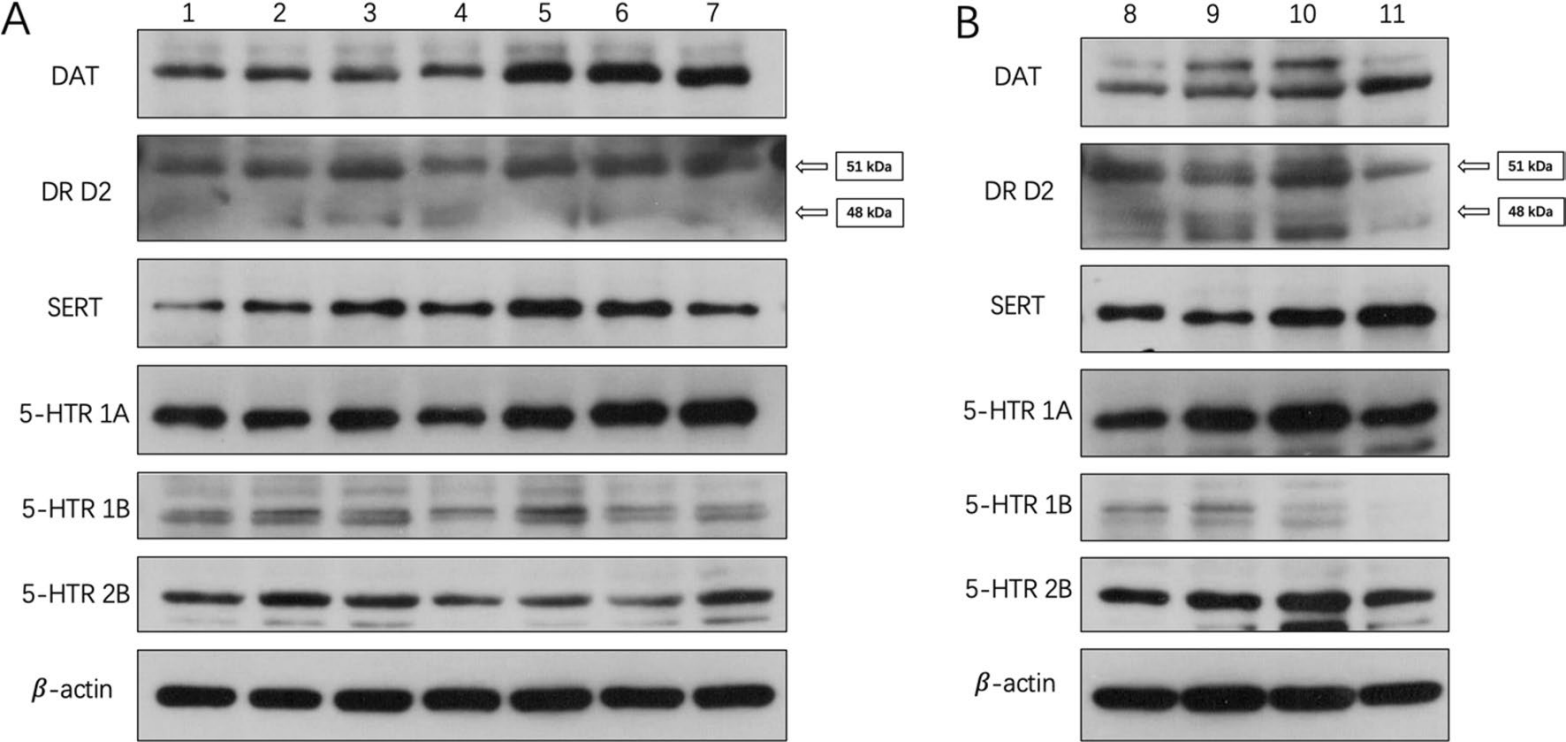
Effects of sulpiride and clozapine on the translation levels of functional proteins involved in DAergic and 5-HTergic systems in zebrafish diencephalon. Note: 1. Control, 2. 20 ng/L sulpiride group, 3. 50 ng/L sulpiride group, 4. 100 ng/L sulpiride group, 5. 20 ng/L clozapine group, 6. 50 ng/L clozapine group, 7. 100 ng/L clozapine group, 8. Control, 9. 20 ng/L sulpiride and clozapine mixture, 10. 50 ng/L sulpiride and clozapine mixture, 11. 100 ng/L sulpiride and clozapine mixture. The grouping of blots was first cropped from different gels and then transferred onto PVDF membranes (1:1) prior to hybridisation with antibodies.

Sulpiride-induced abnormal protein levels of SERT, 5-HTR 1A, and 5-HTR 2B in zebrafish diencephalon (*p* < 0.05). Specifically, DR D2 (48 kDa) was down-regulated by 20 ng/L sulpiride, while DR D2 (51 kDa) was up-regulated and down-regulated by 50 ng/L s and 100 ng/L sulpiride, respectively (Figs. 5, 6). Furthermore, sulpiride significantly increased the protein levels of SERT and 5-HTR 2B, while decreasing that of 5-HTR 1A (*p* < 0.05).

Clozapine-induced abnormal protein levels of DAT, DR D2 (51 kDa), SERT, 5-HTR 1A, 5-HTR 1B, and 5-HTR 2B in zebrafish diencephalon (*p* < 0.05). Specifically, all concentrations of clozapine up-regulated DAT, DR D2 (51 kDa), SERT, and 5-HTR 1A. At 20 ng/L, clozapine up- and down-regulated 5-HTR 1B and 5-HTR 2B, respectively; while 50 ng/L clozapine down-regulated 5-HTR 1B and 5-HTR 2B (*p* > 0.05); and 100 ng/L clozapine down- and up-regulated 5-HTR 1B and 5-HTR 2B, respectively (*p* > 0.05) (Figs. 5, 6).

The sulpiride and clozapine mixture significantly affected the protein levels of DAT, DR D2 (51 kDa), DR D2 (48 kDa), SERT, 5-HTR 1A, 5-HTR1B, and 5-HTR 2B in zebrafish diencephalon (*p* < 0.05). Specifically, the mixture up-regulated DAT compared to the control (Figs. 5, 6). DR D2 (51 kDa) (*p* < 0.05) was down-regulated by the 20 ng/L mixture, while DR D2 (48 kDa) was up-regulated by both the 20 ng/L (*p* > 0.05) and 50 ng/L (*p* < 0.05) mixtures. DR D2 (51 kDa) was down-regulated by the 100 ng/L mixture (*p* > 0.05) (Figs. 5, 6). Moreover, the mixture significantly increased the protein levels of SERT, 5-HTR 1A, and 5-HTR2B, while decreasing the expression of 5-HTR 1B (*p* < 0.05) (Figs. 5, 6).

## Discussion

The therapeutic long-term use of psychotropic drugs has resulted in their presence in the environment. Studies have demonstrated that psychiatric drugs occur frequently in wastewater, surface water, and ground water systems, indicating that even after treatment in the municipal wastewater treatment plants (WWTPs), psychiatric pharmaceuticals enter the environment, where they may cause adverse effects on aquatic organisms, even at low concentrations^21–23^. Sulpiride has been detected in the inflow and outflow of multiple sewage treatment plants in Germany, at concentrations ranging from 111–1100 and 110–338 ng/L, respectively^18^. Similarly, clozapine has been found in the influent and effluent of a municipal sewage treatment plant in Porto, Portugal, at concentrations of 150 and 100 ng/L^24^, respectively. In Beijing, China, the concentrations of sulpiride and clozapine in the influent to the sewage treatment system of the Beijing Psychiatric Hospital ranged from 2762–9832 and 5552–12,782 ng/L, respectively; while the concentrations in the effluent ranged from 432–10,833 and 295–8183 ng/L, respectively^25^. These antipsychotic drugs have also been found in surface waters. For example, the concentrations of sulpiride in the surface waters of German cities range from 28–251 ng/L^18^. Among different rivers of Chinese cities, different concentrations of sulpiride have been detected, with a maximum concentration of 200 ng/L and an average concentration of 82.4 ng/L^19^.

In the current study, we investigated the effects of the antipsychotic drugs sulpiride and clozapine on DA and 5-HT signaling pathways in zebrafish brain. The DAergic system in zebrafish brain is mainly located in the telencephalon and diencephalon. 5-HTergic neurons are present in the diencephalon and hindbrain, and those in the diencephalon project fibers that are widely distributed in telencephalon^9,13,15^. 5-HTergic signaling modulates the activity of DAergic systems, and this action plays an important role in determining normal or abnormal behavior^13,26–28^. Because of interfering with DA and 5-HT signaling by blocking DR D2, or 5-HTR 2A and DR D4, we selected sulpiride and clozapine to explore the effects of antipsychotic drugs on the transcription and translation levels of functional proteins of the DAergic and 5-HTergic systems in zebrafish telencephalon and diencephalon.

Sulpiride specifically blocks the DR D2; the activation of DR D2 that are distributed in the presynaptic membrane can negatively regulate DA synthesis and release by inhibiting the key enzyme for DA synthesis, tyrosine hydrogenase (TH). Thus, TH activity is inhibited and the rates of DA synthesis and renewal are reduced. In addition, DR D1 coordinates the function of DR D2 either independently or synergistically, or by antagonism. Therefore, DR D1 modulates DR D2 by a permissive effect. Our results show that sulpiride up-regulates *TH*, *DAT*, *DR D1*, and *DR D2*, while down-regulating *MAO*. The increased protein levels of DR D2 and DAT are compensated for by enhancing the level of DA signaling. Meanwhile, 5-HT regulates the activity of DA neurons as well as the release of DA from the presynaptic membrane in the mesencephalon by acting on 5-HTR 2A of the DA neurons or 5-HTR 2C of the GABA neurons^29,30^. Therefore, sulpiride also indirectly up-regulated 5-HT synthase *TPH* by inhibiting the DA signaling system. Our results show that sulpiride interferes with the DA signaling system, specifically, it increases the activity of the DAergic system by promoting the recycling of 5-HT at the synaptic cleft and the up-regulation of the expression levels for 5-HT receptors. Our study proved this by showing the up-regulation of *SERT*, *5-HTR 1AA*, *5-HTR 1B, 5-THR 2AA*, and *5-HTR 2B* along with the down-regulation of *MAO*; and we also demonstrated the up-regulation of SERT, 5-HTR 1A, 5-HTR1B, and 5-HTR 2B. Furthermore, the expression levels of functional molecules in DA ergic and 5-HTergic systems were higher in the 50 ng/L group compared to those in the 100 ng/L group. The reason for this may be the high-dose sulpiride that produced negative feedback on the activity of DAergic system, thus down-regulating the expression level of functional molecules in the DA and 5-HT systems. Clozapine is well-known to antagonize both 5-HTR 2A and DR D4 with high and low affinities, respectively^31^. 5-HT neurons in zebrafish are mainly in the diencephalon, projecting 5-HTergic fibers widely to telencephalon; and 5-HT receptors in zebrafish telencephalon are mainly distributed in the presynaptic membrane of 5-HTergic fibers or neuronal cell bodies^13^. 5-HTR 2A are distributed in the DA neuron and they regulate the sensitivity of the DA receptor and adjust or modify the activity of phased DA that results from the action potential^32^. The regulatory activity of 5-HTR 2A is accomplished by modulating the tonic release of the DA^29^. Moreover, the 5-HTR 2A receptor has is also involved in the negative feedback regulation of 5-HT neuronal activity and the release of 5-HT^33^. In addition, 5-HTR1 activates the presynaptic membrane, specifically, 5-HTR 1A regulates neuron firing while 5-HTR 1B regulates the release of 5-HT, causing the inhibition of neuronal activity and a decrease in the amount of transmitter released^34^. Thus, clozapine may increase DA synthesis with compensatory up-regulation of DA receptors and transporter. These effects are accompanied by increased 5-HT signaling, which we have proven by showing the up-regulated mRNA expression levels of *TH*, *DR D1*, *DR D2*, *TPH*, *SERT*, *5-HTR 1AA*, *5-HTR 1B*, *5-THR 2AA*, and *5-HTR 2B* and the down-regulation of *MAO*, with increased protein levels of DAT, DR D2, SERT, 5-HTR 1A, 5-HTR 1B, and 5-HTR 2B. Moreover, the down-regulation of *DAT*, *DR D1*, *5-HTR 1B*, and *5-HTR 2B* by 100 ng/L clozapine is likely due to the adverse effects of this dosage appearing early, resulting in a high number of regulatory molecules being expressed in the early stages of drug exposure. Our results also show that the mixture of sulpiride with clozapine may simultaneously interfere with both the DA and 5-HT systems in zebrafish telencephalon. The 20 ng/L mixture up-regulated *TH*, *DR D1*, and *DR D2*, while down-regulating *MAO*; and it also up-regulated DAT and DR D2, resulting in improved DA system activity mainly through the increase in DA synthesis and transport, and the up-regulation of DA receptor levels, accompanied by the enhanced activity of the 5-HT system, as shown by the up-regulation of *TPH*, *SERT*, *5-HTR1AA*, *5-HTR1B*, *5-THR2AA*, and *5-HTR2B* transcriptional levels and the up-regulation of 5-HTR 1A, 5-HTR1B, and 5-HTR2B. It is notable that the mixture affected the DA and 5-HT systems less than either of the antipsychotic drugs alone, suggesting an antagonistic relationship between sulpiride and clozapine. Moreover, the adverse effects of the mixtures on DAergic and 5-Htergic systems in zebrafish telencephalon declined as their concentrations increased, i.e., the abnormal high expression levels of the functional molecules of the two neurotransmitter systems compared to the control decreased as the concentrations of the drugs decreased, implying that the antagonistic effect between sulpiride and clozapine increased with drug concentration, and the effect on the DA and 5-HT systems in zebrafish telencephalon decreased along with drug concentration. In addition, the 20 ng/L mixture had opposite effects on the transcription and translation of *DAT*, because the organism responded to the adverse effects induced by the mixture early, which also occurred in the 100 ng/L clozapine treatment. This further indicates that sulpiride and clozapine interact antagonistically, as the effect of the low-dose mixture on Daergic and 5-Htergic systems in zebrafish telencephalon was greater than that of the high-dose mixture.

A large number of DA and 5-HT neurons have fibers that are distributed in zebrafish diencephalon, and this plays an important physiological function^7^. Antipsychotic drugs can modulate DA and 5-HT signaling by specifically interfering with receptors in the Daergic and/or 5-Htergic system. We found that sulpiride decreases the synthesis and increases the transport of DA in the synaptic cleft of the zebrafish diencephalon, and this is accompanied by the down-regulation of DA receptor levels, as well as the decreased synthesis and increased transport of 5-HT. This is supported by our observations of the down-regulation of *TH*, *DR D2*, *MAO*, and *TPH*, along with the up-regulation of *DAT*, as well as the up-regulation of SERT and 5-HTR 2B. DR D2 is an autoreceptor that is distributed in the DA neurons and axon terminals, thus, it modulates the firing activity of DA neurons and, by a negative feedback mechanism, regulates DA synthesis and release. Furthermore, 5-HT regulates DA neuron activity and DA release by acting on 5-HTR 2A. Hence, sulpiride may cause abnormal firing of DA neurons or excessive release of DA, resulting in increased levels of DA in the synaptic cleft. These levels induce the organism to regulate DA by reducing its synthesis, increasing its transport in the synaptic cleft, and inhibiting its release (induced by 5-HT). It is clear that clozapine exerts a pharmacological effect by specifically antagonizing 5-HTR2A and DR D4. We found that both 20 and 50 ng/L clozapine up-regulated *TH*, *DR D2*, and *SERT*, while *DAT*, *DR D1*, *TPH*, *5-HTR 1AA*, *5-HTR 1B*, *5-THR 2AA*, *5-HTR 2B*, and *MAO* were down-regulated; and DAT, DR D2 (51 kDa), and SERT were up-regulated, implying that 20 ng/L and 50 ng/L clozapine mainly impacted DA signaling in the diencephalon. Specifically, the activity of DA neurons was decreased, which up- and down-regulated *TH* and *MAO*, respectively, resulting in increased DA levels. Furthermore, DAT was up-regulated due to positive feedback of increased DA levels. Meanwhile *TPH* and *SERT* were down- and up-regulated, respectively, resulting in the inhibition of the 5-HT effect on DA release. In addition, clozapine decreased *DR D1* and increased *DR D2* mRNA expression levels. Moreover, at a concentration of 100 ng/L, clozapine down-regulated *DAT*, *DR D1*, *DR D2*, *SERT*, *5-HTR 1AA*, *5-HTR 1B*, *5-THR 2AAI,* and *5-HTR 2B*, while up-regulating *TPH* and *MAO*, along with DAT, DR D2 (51 kDa), and SERT, indicating that high-dose clozapine mainly inhibits the negative feedback control of 5-HT release. Therefore, *TPH* up-regulation along with *SERT*, *5-HTR1AA*, and *5-HTR1B* down-regulation occurs to increase the level of 5-HT in the synaptic cleft, which interferes with 5-HT signaling in the diencephalon. At 20 ng/L, the mixture down-regulated *TH*, *DR D1*, *DR D2*, *SERT*, *5-HTR 1AA*, *5-HTR 1B*, and *5-THR 2AA*, while up-regulating *DAT*, *TPH*, *5-HTR2B*, and *MAO*, along with DAT and DR D2 (48 kDa), which indicates that sulpiride induces an abnormal increase in DA levels at the synaptic cleft, resulting in both decreased DA synthesis and increased DA transport from the synaptic cleft. Meanwhile, clozapine inhibits the negative feedback regulation of 5-HT release, resulting in increased 5-HT synthesis and decreased 5-HT transport from the synaptic cleft. The effects of the 50 ng/L mixture on the DAergic and 5-HTergic systems were similar to those of the 20 ng/L mixture, except the clearance of excess 5-HT in the synaptic cleft was through SERT transport in the 50 ng/L mixture, while the process was mediated by MAO in the 20 ng/L mixture (which was proven by the down-regulation of *MAO* and up-regulation of *SERT*). Similarly, the 100 ng/L mixture, the antipsychotics interfered with DA and 5-HT signaling at an earlier stage compared to that in the 20 and 50 ng/L mixtures. Therefore, much of the up- or down-regulation of *DAT*, *TPH*, and *SERT* occurred earlier, which was likely due to the high concentrations of the antipsychotic drugs. These results show that the mixture of sulpiride and clozapine impacts DAergic and 5-HTergic systems in zebrafish diencephalon.

Because of their resistance to degradation, pharmaceuticals are the most frequently detected emerging contaminants in the aquatic environment^35^. This persistence, along with their physiological activity, means that these compounds pose a long-term risk to aquatic ecosystems. Antipsychotic drugs are among the most prescribed active drugs in the world^36^, and their use has increased significantly because of the COVID-19 global pandemic^37–39^.The results of the present study suggest that antipsychotic drugs sulpiride and clozapine interfere with DAergic and 5-HTergic neurotransmitter systems in zebrafish telencephalon and diencephalon, causing abnormal regulation of the expression levels of functional molecules involved in DA and 5-HT signaling. DAergic and 5-HTergic systems play an important role in regulating brain functions such as movement, motivation, behavior, learning, memory, and emotions, therefore, by interfering with the DA and 5-HT signaling in brain, sulpiride and clozapine may impact these physiological functions and seriously threaten the health of zebrafish.

## Conclusion

The results of our investigation on the effects of environmentally-relevant concentrations of antipsychotic drugs on DAergic and 5-HTergic systems in zebrafish brain suggest that sulpiride and clozapine affect the normal regulation of the expression levels of functional molecules involved in DA and 5-HT signaling. These drugs interferes with DAergic and 5-HTergic systems and seriously threatens the health of zebrafish. Our results indicate that further study is needed to determine the effects of antipsychotic drugs on the physiological function of the zebrafish brain.

## Supplementary Information

Supplementary Information 1.

Supplementary Information 2.

## Data Availability

All data generated or analyzed during this study are included in this published article.
