# Supplementary figures and images for: Environmentally-relevant concentrations of the antipsychotic drugs sulpiride and clozapine induce abnormal dopamine and serotonin signaling in zebrafish brain
Source: Sci Rep. 2022 Oct 26;12:17973. doi: 10.1038/s41598-022-22169-1 (PMC9606268; doi:10.1038/s41598-022-22169-1)

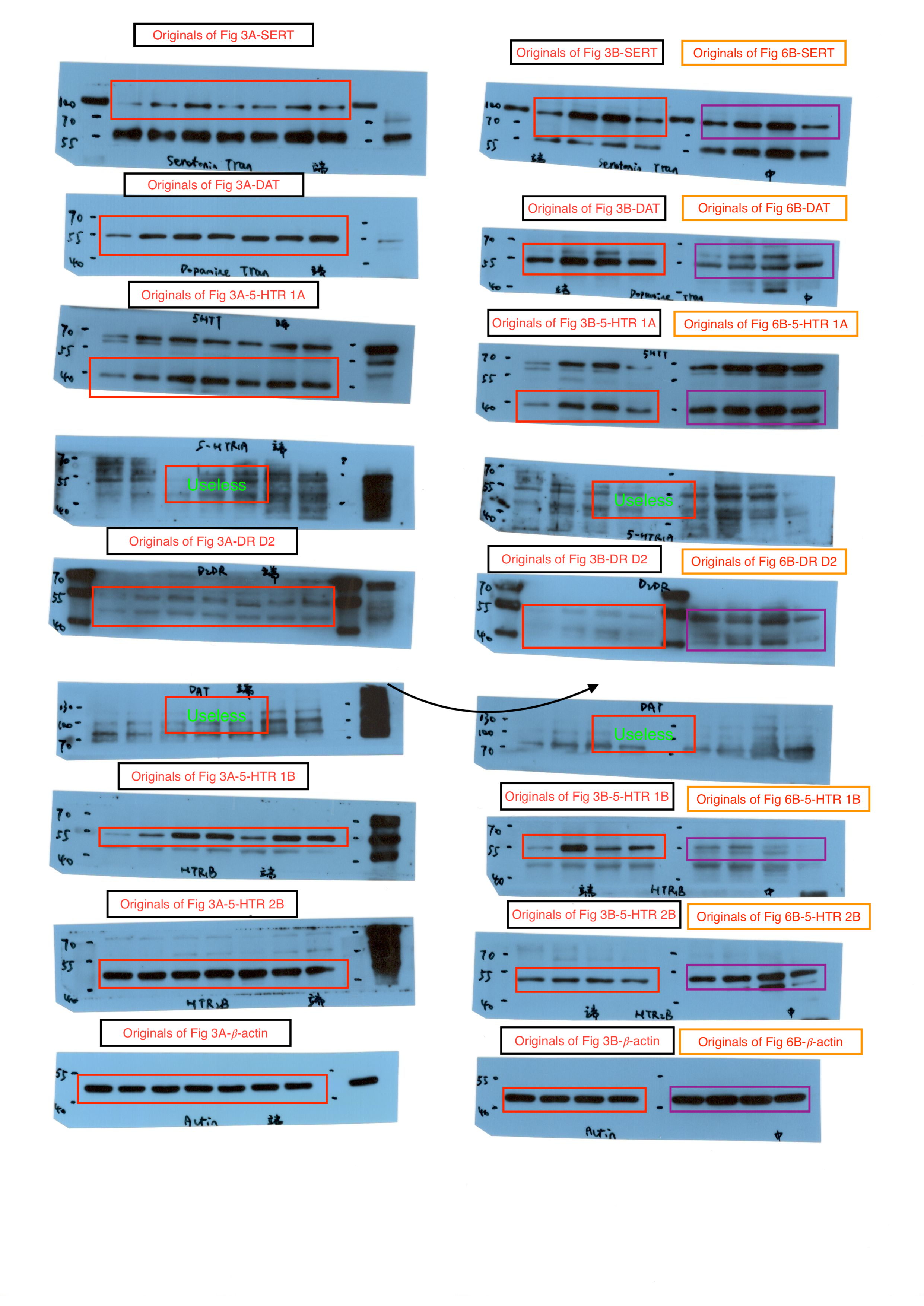

Supplement: Supplementary file 1 — Supplementary Information 1. [file 41598_2022_22169_MOESM1_ESM.tif]

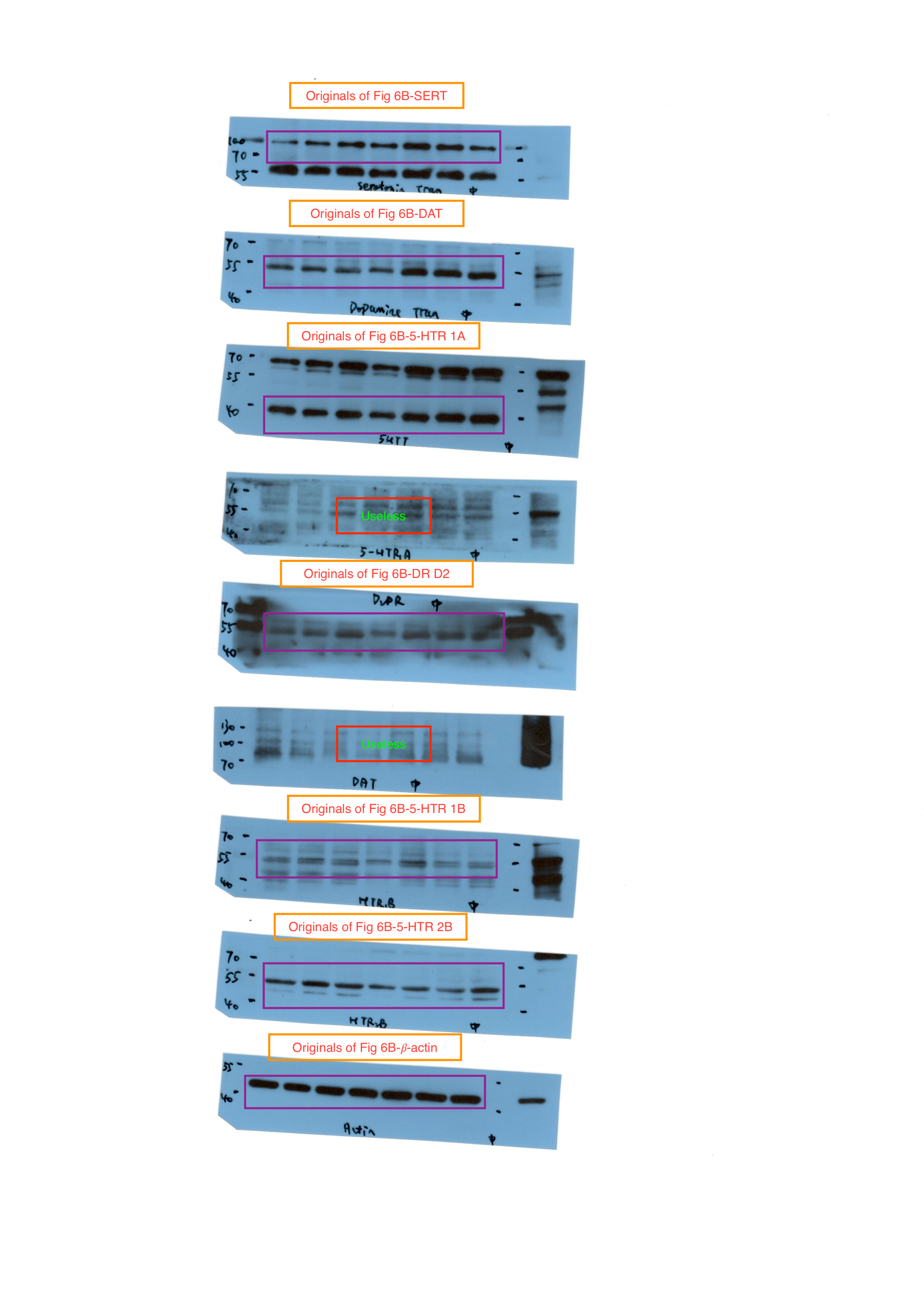

Supplement: Supplementary file 2 — Supplementary Information 2. [file 41598_2022_22169_MOESM2_ESM.tif]
